# The Effectiveness of Electroacupuncture for Functional Constipation: A Randomized, Controlled, Clinical Trial

**DOI:** 10.1155/2015/670963

**Published:** 2015-05-03

**Authors:** Nili Da, Xinjun Wang, Hairong Liu, Xiuzhu Xu, Xun Jin, Chaoming Chen, Dan Zhu, Jiejing Bai, Xiaoqing Zhang, Yangyang Zou, Guangyong Hu, Jianbin Zhang

**Affiliations:** ^1^Second Clinic Medical School, Nanjing University of Chinese Medicine, Nanjing 210000, China; ^2^Department of Acupuncture, People's Hospital of Jurong, Road 60, West Street of Huayang Town, Jurong, Zhenjiang 212400, China; ^3^Anorectal Department, Third Affiliated Hospital of Nanjing University of Chinese Medicine, Nanjing, China

## Abstract

*Background*. Electroacupuncture (EA) has been reported to treat functional constipation (FC). The aim of this study was to investigate the efficacy and safety of EA with different needle insertion method for FC. *Methods*. Sixty-seven participants were randomly assigned to control (EA with shallow puncture) and EA (with deep puncture) groups. Every patient received 5 treatments per week in the first two weeks, then 3 treatments per week during the following six weeks. Complete spontaneous bowel movements (CSBM), spontaneous bowel movements (SBM), Bristol stool scores (BSS), and Patient Assessment of Constipation Quality of Life (PAC-QOL) were assessed. *Results*. Both shallow and deep EA significantly increased CSBM frequency compared to the baseline. CSBM was increased from 0.50 ± 0.59/wk to 2.00 ± 1.67/wk with deep EA and from 0.48 ± 0.59/wk to 1.33 ± 1.09/wk with shallow EA (*P* < 0.05, resp.). Similar finding was noted in SBM. Deep EA was more potent than shallow EA (*P* < 0.05) during the treatment period. No difference was found on BSS and PAC-QOL between two groups. *Conclusion*. It is effective and safe with EA to treat FC. Studies with large sample size and long-term observation are needed for further investigation.

## 1. Introduction

According to Rome III diagnostic criteria [1], functional constipation (FC) is characterized by hard, infrequent, or incomplete defecation. The prevalence of FC in North America is from 1.9% to 27.2% [2], 7.4% in Mexico [3], and 2.4–11.2% in Iran [4]. In recent years, functional constipation occurs more frequently in China, with total prevalence of 9.18% [5], and in the elderly was 67.87% [6].

Constipation may cause disorders in perianal, such as perianal abscess and anal fistula; anorectal lesions, such as hemorrhoids and colorectal cancer; digestive system diseases, such as bloating, indigestion, and diverticulosis; psychiatric symptoms, such as headache, insomnia, and irritability, aggravating the symptoms, even threatening the life, such as increasing blood pressure, inducing acute cerebral vascular disease, and even sudden death [7, 8]. Constipation also seriously affects the quality of life [9]. It was reported that in 2010 the costs related to hospitalizations of constipation as the primary diagnosis were over 850 million dollars in the US [10]. In addition, patients with constipation were known to have reduced quality of life.

More and more constipation patients prefer alternative and complementary treatment because of worry from drug side effect and deficiency of long-term effect [11], despite laxatives having been widely used. A few studies have reported the effectiveness of acupuncture for treating FC [12, 13]; however, these studies lacked comprehensive study design. Therefore, it is necessary to complete a randomized, controlled, patient blinded, and clinical trial to investigate the efficacy and safety of electroacupuncture treatment of functional constipation.

## 2. Methods

### 2.1. Study Design and Ethics Approval

The recruitment of subjects took place from October 2012 to September 2013. The study was approved by Medical Ethics Committee and completed in the Outpatient Department of Guo Yi Tang in Nanjing, China.

**Figure 1:**
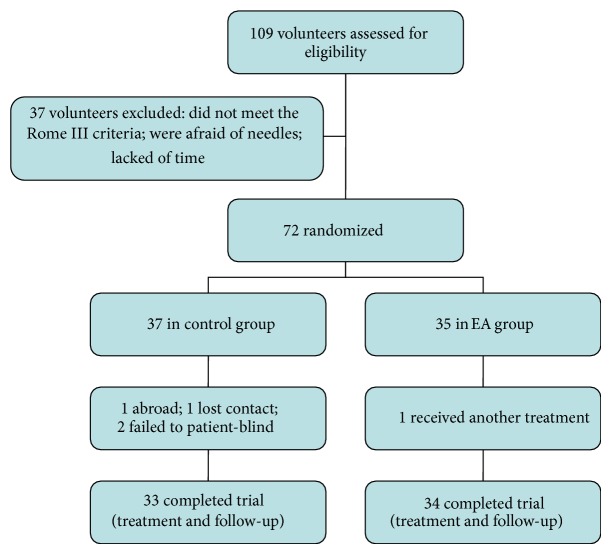
Trail flow chart.

As shown in Figure 1, total 67 patients (13 male and 54 female) with FC were finally enrolled to the experiment. Participants were included if they met all of the following conditions: (1) diagnosed with FC according to the Roman III criteria [1]; (2) aged between 18 and 65 years; (3) CSBM ≤ twice per week at least three months; (4) without any treatments (except rescue methods being used when participants had intolerable discomfort) at least two weeks before joining this study.

Participants were excluded from the study if they had a diagnosis of irritable bowel syndrome (IBS), or constipation caused by other diseases or medicine, or other significant diseases and medicine that may interfere with completion of the study. Pregnant or breastfeeding women were also excluded.

Patients had the rights to decide to whether participate in or withdraw the study at any time. Their decisions did not affect their deserved treatments.

Participants recruited through advertisements in hospitals and schools were randomized by stochastic systems in computer and decided to receive control or EA treatment. All participants were blinded to the type of treatment method received until completion of the study.

### 2.2. Treatments

**Figure 2:**
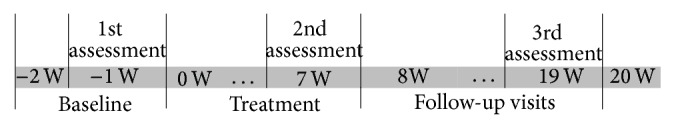
The total study period and the timepoint of evaluation.

The total study period was shown in Figure 2. After two-week baseline assessment, each patient was treated with either deep EA or shallow EA for 8 weeks followed by 12 weeks follow-up period.

Each patient received total 28 treatments, including 5 times per week for the first two weeks and 3 times per week for the following six weeks.

Patients in EA group received EA at 6 acupoints, ST25 (Tianshu) and SP14 (Fujie) and ST37 (Shangjuxu), bilaterally. The physician inserted into ST25 and SP14 with HuaTuo 0.30 × 75 mm needles, deep to the parietal peritoneum without lifting and twisting. The two needles at ST25 and SP14 unilaterally were connected to an electric stimulator (HANS-200A, Nanjing Jisheng Co., China) for 30 min. The frequency was 2/15 Hz alternately. The current was strong enough (0.1 mA–1.0 mA) to produce a slight tremor in patients' abdominal muscles. HuaTuo 0.30 × 40 mm needles were inserted into ST37 with depth of 1 cun, lifted and twisted slightly three times to Deqi every 10 minutes for a total of 30 minutes. Patients in the control group received EA with same techniques and parameters, but with shallow puncture with depth of 2 mm and at points located one cun away from those 6 acupoints (on the median between Stomach Meridian of Foot Yang-ming and Spleen Meridian of Foot Tai-yin), respectively, without lifting and twisting, for 30 minutes.

### 2.3. Assessment

The primary outcome was CSBM (complete spontaneous bowel movements); the secondary outcomes consisted of spontaneous bowel movements (SBM), Bristol stool scores (BSS), hard defecation score, and Patient Assessment of Constipation Quality of Life (PAC-QOL). The participants filled the defecation diary every day during the entire experimental period.

### 2.4. Statistical Analysis

All of statistical analysis was performed in both ITT analysis (intention-to-treat analysis) and PP analysis (per-protocol analysis). The data are expressed as the mean ± standard error (SEM) in each group. SPSS Win. Ver.14.0 software was used and *P* < 0.05 was considered as significance.

## 3. Results

### 3.1. Outcomes

One hundred and nine volunteers were filtered in this study, and 37 volunteers were excluded due to either failure to meet the Rome III criteria or being afraid of needle insertion or lacking of time to complete the experiment. Then 72 participants were divided into control group (*n* = 37) or EA group (*n* = 35) randomly; 67 participants completed all treatments and the follow-up visits. In control group, two participants lost contact, and the other two failed in blinding. One participant in EA group received another treatment of constipation (Figure 1).

At the 1st assessment (baseline, before treatment), there were no significant differences between the two groups, including gender, age, and disease course (Table 1).

**Figure 3:**
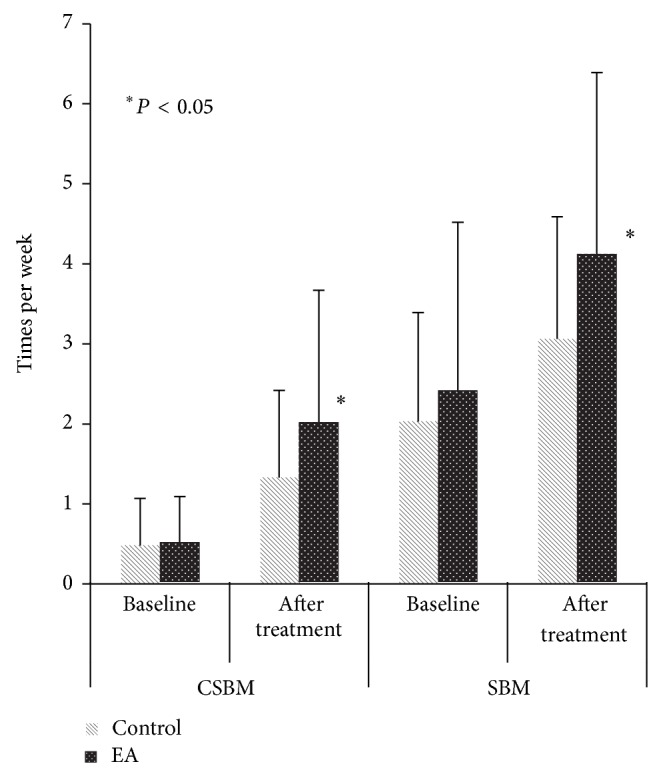
CSBM and SBM (mean ± SD).

At the 2nd assessment (after treatment of 8 weeks), CSBM and SBM were increased significantly in EA group (*n* = 34, 2.00 ± 1.67/week and 4.10 ± 2.29/week, resp.), compared to control group (*P* < 0.05, *n* = 33, 1.33 ± 1.09/week and 3.06 ± 1.53/week, resp., Figure 3). However, at the 3rd assessment (follow-up visits of 12 weeks), there was no difference between the two groups on CSBM (data not supplied).

**Figure 4:**
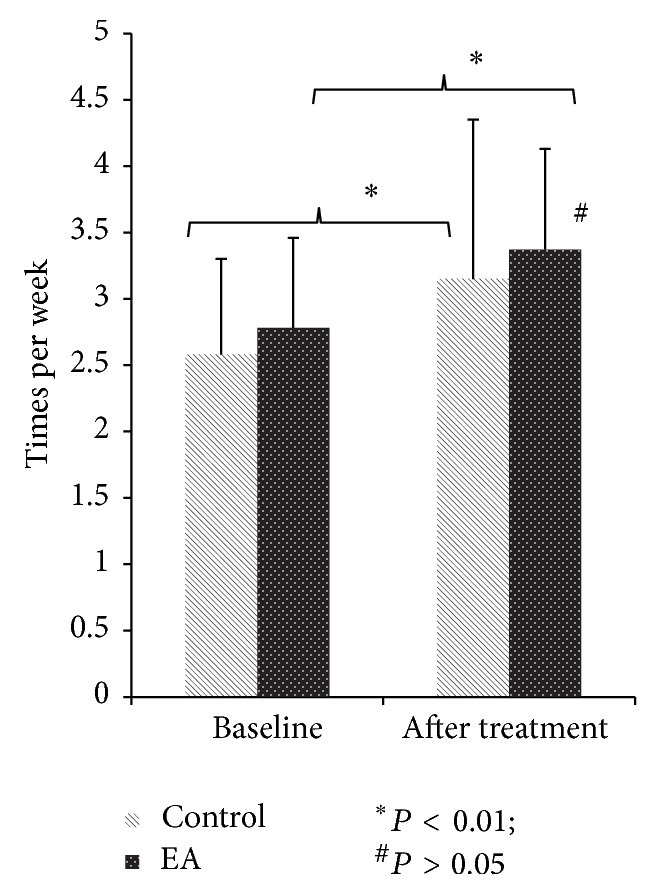
BSS (mean ± SD).

**Figure 5:**
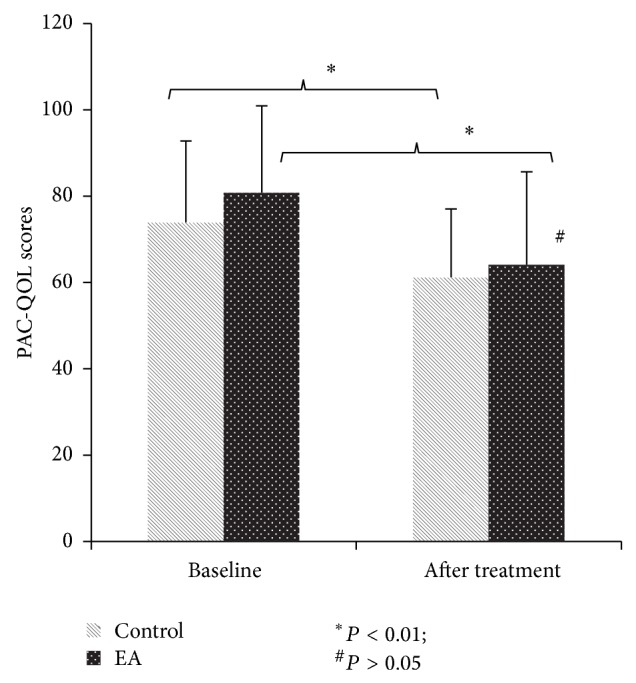
PAC-QOL score (mean ± SD).

Both treatment methods significantly increased BSS and PAC-QOL compared to the baseline (*P* < 0.01, resp.); however, no differences were found between the two treatment methods (*P* > 0.05) (Figures 4 and 5).

According to Rome III criteria, we consider CSAM≧3 as a standard indicating the success of treatment. The cure rate of EA group was higher than that in control group (*P* = 0.014) (Table 2).

### 3.2. Safety

There were no serious adverse events reported. Local subcutaneous congestion appeared in two participants; one participant reported mild abdominal pain.

## 4. Discussion

Electroacupuncture (EA) is based on acupuncture, an ancient Chinese traditional medicine therapy, in which electric current is transmitted to needles inserted acupoints on skin. During the past decade, EA has been reported to treat constipation by acupuncturists. However, evidences to efficacy and safety are deficiency because of less randomized controlled clinic trails reported.

In this study, EA showed effective on constipation. Times of spontaneous bowel movements per week were increased; properties of stool were improved so that evacuation became smooth; qualities of life of patients with constipation were taking a turn for the better.

Nonacupoints were active in control group, despite the fact that they locate at one cun away from normal acupoints and the middle of two meridians. In the literature, opinions on nonacupoints were controversial, especially the distance between nonacupoint and normal acupoint. Some researchers consider that acupoint is not located at a point on skin but in a field [14]; therefore the more proper name of acupoint is “acupuncture field” [15]. Moisberger recommend “a minimum distance of 6 cm between verum and sham points on face, hands and feet, and up to 12 cm for all other parts of the body” [15]. However, this is not feasible because there are so many acupoints throughout the body; it is understandable that all acupoints interfere with each other within the distance of 6 cm or 12 cm. In the current study, although using the shallow needle insertion, the control group also received EA treatment and therefore improved defecation frequency and constipation symptom scores.

The technique of deep puncture performed on acupoints ST25 and SP14 caused that EA group acted better than control group. Taking needles perpendicularly and slowly into skin of abdomen until penetrating the peritoneum had been proved effective for constipation [16]. Operative technique of puncture is deemed to be one of important factors which can affect acupuncture action. So the direction and depth of puncture should be required. Needles penetrated the peritoneum, stimulated intestine directly, and improved motility and at the same time avoided impairing organs due to without lifting and twisting. The safety of “deep acupuncture” on ST25 had been confirmed through study of anatomy and operation standard had been set up [17]. No obvious adverse events have been noted in the current study.

The mechanism of EA for treating constipation could be attributed to the improvement of colonic motility. It was reported that EA promotes contractility of distal colon in rats [18]. EA was also shown to accelerate colon motility and transit in rats [19]. Rectal distention, a common model to mimic feces stasis, has been shown to alter gastric slow waves and delay gastrointestinal transit. Using the rectal distention model, EA was shown to normalize the impaired gastric slow waves and improve antral contractions in dogs and improve upper and lower abdominal symptoms in healthy volunteers [20, 21]. These effects are believed to be mediated via cholinergic and opioid pathways [18–21].

In conclusion, it is effective and safe with EA to treat FC. There are deficiencies in this study, including small sample sizes and single blind. More rigorous studies with larger sample sizes are required.

## Figures and Tables

**Table 1:** Patients demographics (mean ± SD).

	Control (*n* = 33)	EA (*n* = 34)	*P*
Sex (female (%))	81.82%	79.41%	0.803
Age (years)	37.00 ± 17.89	37.94 ± 18.06	0.768
Course (months)	106.21 ± 91.98	139.59 ± 112.68	0.289

**Table 2:** The cure rate.

	*n*	Cured	Not cured	Cure rate	*P*
Control	33	1	32	3.03%	0.014
EA	34	8	26	23.53%	
